# 581. Enhancing Diversity, Equity, Inclusion, Access, and Justice (DEIAJ) Teaching in Infectious Diseases: Current State and Future Directions in Faculty Development

**DOI:** 10.1093/ofid/ofae631.176

**Published:** 2025-01-29

**Authors:** Hailee Nielsen, Emily Abdoler

**Affiliations:** University of Michigan Medical School, Ann Arbor, MI; University of Michigan, Ann Arbor, MI

## Abstract

**Background:**

Infectious Diseases (ID) clinicians occupy a unique position at the interface of clinical medicine and social justice. Medical education in ID therefore presents a rich opportunity for trainees to cultivate critical consciousness in Diversity, Equity, Inclusion, Access, and Justice (DEIAJ). Research has focused on improving DEIAJ education for ID fellows and attendings (Gleeson et al., 2023, Vasishta et al., 2023 ), but little is known about ID faculty members’ perceptions of DEIAJ teaching. We aim to explore ID faculty members’ attitudes and experiences discussing DEIAJ topics when teaching clinically.

**Figure:**
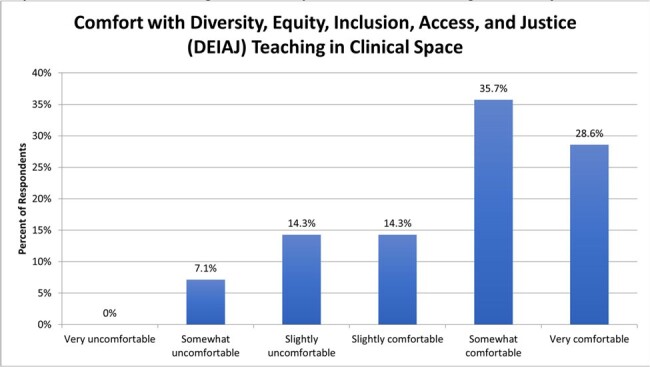

**Methods:**

Clinical ID attendings at the University of Michigan were invited to complete an anonymous survey. Open-ended questions assessed which ID-related DEIAJ topics faculty members believe are most important for students to learn. Likert-style questions evaluated comfort and preparedness regarding DEIAJ teaching, while multiple choice questions explored barriers to covering this content and faculty development preferences.

**Figure:**
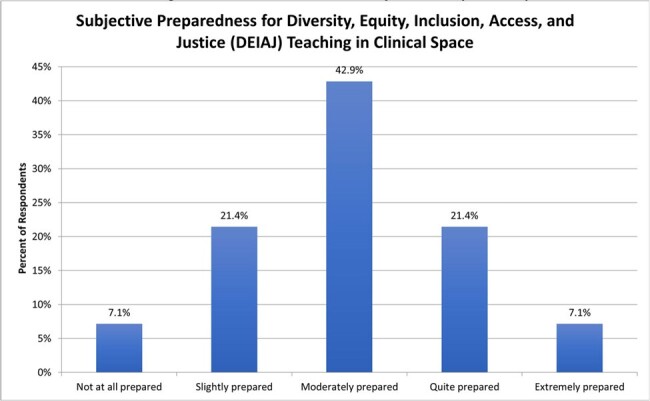

**Results:**

Survey response rate was 81% (22/27), with a 63% completion rate. Faculty comfort with DEIAJ teaching was high, while reported preparedness was only moderate (Figures 1, 2). Common perceived barriers to DEIAJ teaching were lack of facilitation skills (50%) and fear of trainee criticism (42%). Preferred modes for faculty development were DEIAJ integration in conferences (85%), facilitation training (57%), and access to DEIAJ teaching cases (50%). Respondents identified common and important DEIAJ teaching topics that arise in ID clinical practice (Figure 3).

**Figure:**
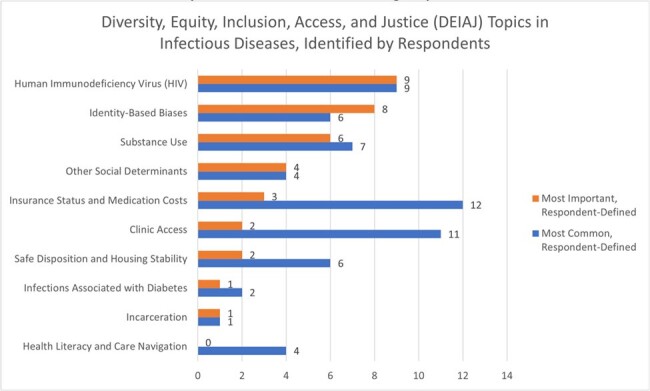

**Conclusion:**

This single-institution study reveals that ID attendings are comfortable addressing DEIAJ topics in their clinical teaching despite not feeling optimally prepared. Identifying this comfort-preparation gap in our division, as well as preferred faculty development modalities and prioritized DEIAJ topics within ID, is the first step in creating integrated professional development opportunities that capitalize on faculty members’ dedication to DEIAJ teaching. Exploration of this issue on a national scale could support development of a customizable professional development toolkit for the broader ID community.

**Disclosures:**

**All Authors**: No reported disclosures

